# Occluding morphologically complicated left atrial appendage using the small-umbrella LAmbre device

**DOI:** 10.1186/s12872-022-02770-y

**Published:** 2022-07-23

**Authors:** Hong-Da Zhang, Ming Yang, Yang-Bo Xing, Si-Xian Weng, Lei Ding, Xiao-Tong Ding, Li-Xing Hu, Ying-Jie Qi, Feng-Yuan Yu, Jing-Tao Zhang, Pi-Hua Fang, Wei Hua, Shu Zhang, Min Tang

**Affiliations:** 1 Arrhythmia Center, State Key Laboratory of Cardiovascular Disease, Fuwai Hospital, National Center for Cardiovascular Diseases, Chinese Academy of Medical Sciences & Peking Union Medical College, 167 Beilishi Road, Xicheng District, Beijing, 100037 China; 2 Department of Cardiology, The People’s Hospital of Yuxi City, The 6th Affiliated Hospital of Kunming Medical University, Yuxi, 653100 Yunnan China; 3 Department of Cardiology, Shaoxing People’s Hospital, Shaoxing, 312000 Zhejiang China

**Keywords:** Left atrial appendage occlusion, Atrial fibrillation, LAmbre, Small-umbrella, Thrombosis

## Abstract

**Background:**

Percutaneous left atrial appendage (LAA) occlusion is effective for stroke prevention in patients with atrial fibrillation. LAA can have a complex anatomy, such as multiple lobes or a large orifice, which may render it unsuitable for occlusion using regular devices. We aimed to investigate the feasibility, safety, and short-term efficacy of the small-umbrella LAmbre device for morphologically complicated LAA.

**Methods:**

We retrospectively enrolled 129 consecutive patients who underwent LAA occlusion using the LAmbre device; the small-umbrella LAmbre device was used in 30 of these patients. We analyzed patients’ characteristics, procedural details, and outcomes.

**Results:**

Twenty-two patients (73.3%) had multilobed (≥ 2) LAA. The umbrella of the occluder was anchored in the branch in 9 patients and in the common trunks of branches in 13 patients. The landing zone and orifice diameters were 19.0 ± 4.39 mm and 27.4 ± 3.95 mm, respectively. The sizes of the umbrella and occluder cover were 22.0 ± 3.42 mm and 34.3 ± 2.75 mm, respectively. At 3-month follow-up transesophageal echocardiography in 24 patients, no peri-device residual flow was reported. Device thrombosis was detected in one patient at 3 months and disappeared after 3 months of anticoagulation. Ischemic stroke occurred in one patient; no other adverse events were reported.

**Conclusions:**

Occlusion of morphologically complicated LAA using the small-umbrella LAmbre device was feasible, safe, and effective in patients with atrial fibrillation in this study. This occluder provides an alternative for patients who cannot be treated with regular-sized LAA occlusion devices.

**Supplementary Information:**

The online version contains supplementary material available at 10.1186/s12872-022-02770-y.

## Introduction

Atrial fibrillation (AF) was first recognized as a risk factor for systemic embolism, especially stroke, in patients with rheumatic valvular disease [1–5]. Decades later, the Framingham study demonstrated that patients with non-valvular AF (NVAF) also had a greater risk of stroke than those without AF [1, 2, 6–8]. Transesophageal echocardiography (TEE) studies have since revealed that more than 10% of patients with NVAF exhibit left atrial thrombus, more than 90% of cases of which are located in the left atrial appendage (LAA) [9]. Based on these findings, percutaneous LAA occlusion has been investigated and has proven to be safe and effective for stroke prevention in patients with NVAF [1, 2, 10–16].

To date, more than 10 commercially available LAA occlusion devices have been approved in North America, Europe, and Asia [10, 17]. The most widely used are the plug type, including WATCHMAN (Boston Scientific), and the pacifier type, including Amulet (Abbott Vascular), Ultraseal LAA Occluder (Cardia), and LAmbre (Lifetech) [4, 17]. Many of the occlusion devices mentioned above are designed for single-lobe LAA and only come in limited sizes; thus, they are limited in their use for occlusion of LAA with a complicated anatomy, especially LAA with multiple lobes [17]. In contrast, the LAmbre occluder, which is a pacifier-type device, consists of an inner umbrella and an outer cover. The LAmbre occluder comes in 11 regular sizes for single-lobe LAA and 6 special sizes with a small umbrella and a large cover for multi-lobe LAA [17]. LAA closure with the LAmbre device showed encouraging results in previous studies [15, 18–20]. Surprisingly, the small-umbrella LAmbre device was underused in those studies, and there are only a couple of case reports showing its effectiveness [21–23]. In this study, we aimed to evaluate the feasibility, safety, and short-term efficacy of the small-umbrella LAmbre device for occlusion of morphologically complicated LAA in patients with NVAF.

## Methods

### Study population

This was a retrospective multi-center study. Between September 2017 and May 2021, 129 patients at three hospitals in China underwent LAA occlusion using the LAmbre device, and the small-umbrella LAmbre device was used in 30 of these patients.

The primary enrollment criteria included: (1) NVAF; (2) age ≥ 18 years; and (3) CHA_2_DS_2_-VASc score ≥ 2 (≥ 3 for females). Patients were also required to meet at least one of the following three conditions: (1) not suitable for long-term anticoagulation (contraindications to anticoagulation or documented poor adherence to anticoagulation and refusal to undergo anticoagulation even after personal and detailed advice); (2) stroke events when taking adequate anticoagulation therapy with evidence of thrombosis originating from the LAA; (3) HAS-BLED score ≥ 3.

Morphologically complicated LAA was defined as one of the following: (1) multi-lobe LAA; (2) single-lobe LAA with a small tubular body and a large orifice (see more in the Discussion Section).

All patients underwent transthoracic echocardiography and TEE to exclude intracardiac thrombosis, including LAA thrombosis 48 h before LAA occlusion [10]. Further exclusion criteria included other comorbidities that required anticoagulation, other comorbidities that required cardiac surgery, acute myocardial infarction or unstable angina, symptomatic carotid disease, hemorrhagic disease, presence of a prosthetic valve, severe valvular disease, left ventricular ejection fraction < 30%, New York Heart Association functional class IV, ≥ moderate pericardial effusion, estimated survival < 1 year, recent stroke or transient ischemic attack within 30 days, pregnancy, and infective endocarditis.

This study was performed in accordance with the Declaration of Helsinki and was approved by Ethics Committee of Fuwai Hospital (Approval Number: 2021–1575) on 06 December 2021. Informed consent was obtained from all participants.

### Device implantation

**Fig. 1:**
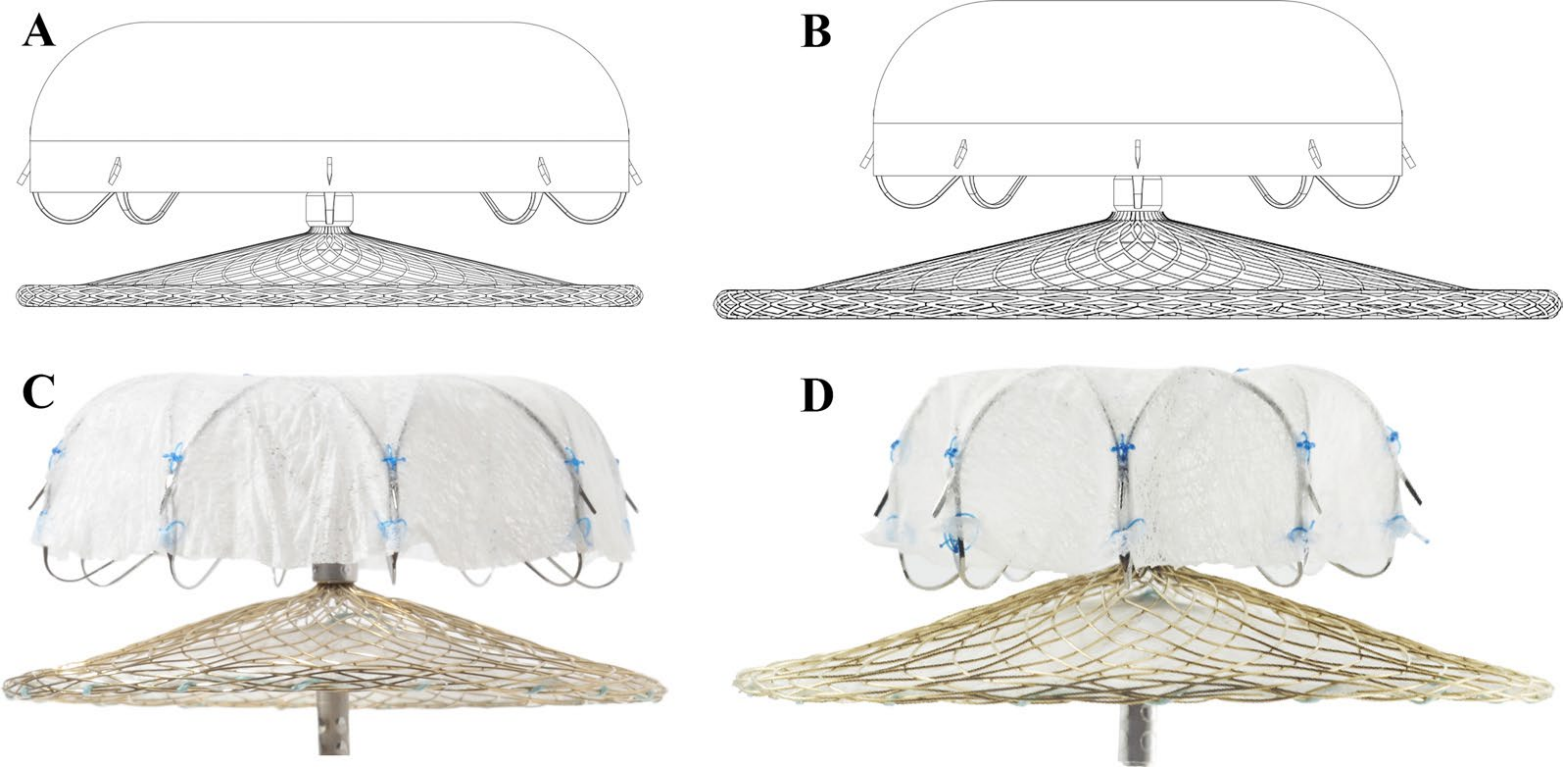
The LAmbre LAA occlusion device. The schematic diagrams (**A** and **B**) and real-life products (**C** and **D**) of the LAmbre occlusion device. The upper part is the inner umbrella anchored inside the LAA. The lower part is the outer cover placed on the LAA orifice. The umbrella and the cover are connected by a short, flexible central waist. **A** and **C** The regular-sized device with a cover diameter 4–6 mm greater than the umbrella. **B** and **D** The small-umbrella device with a small umbrella and a large cover (diameter difference of 12–14 mm). LAA: left atrial appendage

The LAmbre LAA occlusion system and the implantation procedure have been described in previous studies [15, 20]. Implantation was guided by angiography and TEE under general anesthesia or by intracardiac echocardiography (ICE) under local anesthesia. For the regular-sized LAmbre device, the diameter of the cover was 4–6 mm greater than the umbrella (Fig. 1A and C). By contrast, the diameter of the cover was 12–14 mm greater than the umbrella for the small-umbrella device (Fig. 1B and D). Size selection was based on the anatomy of the LAA (angiographic measurements), and a final decision was made after discussion between at least two experienced operators.

Major periprocedural complications included death, pericardial effusion requiring intervention, stroke, major bleeding, device dislocation requiring surgery, and access vessel complications requiring intervention.

### Treatment after LAA occlusion

After the procedure, patients underwent anticoagulation within 3 months, dual antiplatelet therapy at 3–6 months, and long-term mono-antiplatelet therapy 6 months after the procedure. The course of anticoagulation was extended in patients with stroke events or thrombosis formation on the device.

### Follow-up

Three months after the procedure, a repeat TEE was performed. At 6 months, 1 year, and every year thereafter, follow-up contrast cardiac computed tomography (CT) or TEE was performed in cases of suspected thrombosis.

The primary efficacy endpoint was successful device implantation and successful sealing of the LAA, as measured by TEE at 3 months after the procedure. Successful sealing was defined as no peri-device residual flow or < 3 mm of residual flow, as measured by TEE at 3 months after the procedure.

Major adverse events during follow-up included all-cause death, cardiovascular death, unexplained death, stroke, systemic embolism, device thrombosis, device dislocation, and serious pericardial effusion.

### Statistical analysis

Continuous variables are expressed as mean ± standard deviation or median (interquartile range), as appropriate, and categorical parameters are expressed as ratio or percentage. Data analyses were performed using R version 4.0.2.

## Results

### Baseline characteristics of patients

The baseline clinical characteristics of all 30 patients are presented in Table 1. The mean age of patients was 66.8 ± 9.9 years, and 20 patients (66.7%) were male. Twenty-six patients had persistent AF (86.7%), and 4 patients had paroxysmal AF (13.3%). The most prominent comorbidity was hypertension (76.7%), and half of the patients had a history of transient ischemic attack or stroke. Heart failure (36.7%), diabetes mellitus (30%), and coronary artery disease (26.7%) were also common comorbid conditions. The mean CHA_2_DS_2_-VASc and HAS-BLED scores were 4 ± 1.6 and 3 ± 1.0, respectively. The mean left atrial diameter was 42 ± 6.7 mm, and the mean left ventricular ejection fraction was 58 ± 9.4%.

**Table 1:** Baseline characteristics of all patients (n = 30)

Demographics	
Age, yrs	66.8 ± 9.9
Age ≥ 65 yrs	17 (56.7)
Age ≥ 75 yrs	6 (20)
Male sex	20 (66.7)
BMI, kg/m^2^	24.9 ± 3.92
AF type	
Paroxysmal	4 (13.3)
Persistent	26 (86.7)
Comorbidities	
Hypertension	23 (76.7)
Coronary artery disease	8 (26.7)
Prior PCI/CABG	2 (6.7)
Congestive heart failure	11 (36.7)
Diabetes mellitus	9 (30.0)
Previous TIA or Stroke	15 (50.0)
Peripheral arterial disease	3 (10.0)
Echocardiography parameters	
Left atria dimension (AP), mm	42 ± 6.7
LVEDD, mm	48 ± 5.1
Ejection fraction, %	58 ± 9.4
CHA_2_DS_2_-VASc score	4 ± 1.6
1	0 (0)
2	7 (23.3)
3	6 (20.0)
4	4 (13.3)
5	6 (20.0)
6	5 (16.7)
7	2 (6.7)
HAS-BLED score	3 ± 1.0
1	0 (0)
2	10 (33.3)
3	11 (36.7)
4	8 (26.7)
5	1 (3.3)

*AF* atrial fibrillation, *AP* anteroposterior, *BMI* body mass index, *CABG* coronary artery bypass graft, *CHA*_*2*_*DS*_*2*_*-VASc* congestive heart failure, hypertension, age ≥ 75 years, diabetes mellitus, stroke, vascular disease, age 65–74 years, sex category (female), *HAS-BLED* hypertension, abnormal renal/liver function, stroke, bleeding history or predisposition, labile international normalized ratio, elderly (> 65 years of age), concomitant drugs/alcohol, *LVEDD* left ventricular end-diastolic dimension, *PCI* percutaneous coronary intervention, *TIA* transient ischemic attack

### Procedure details

**Fig. 2:**
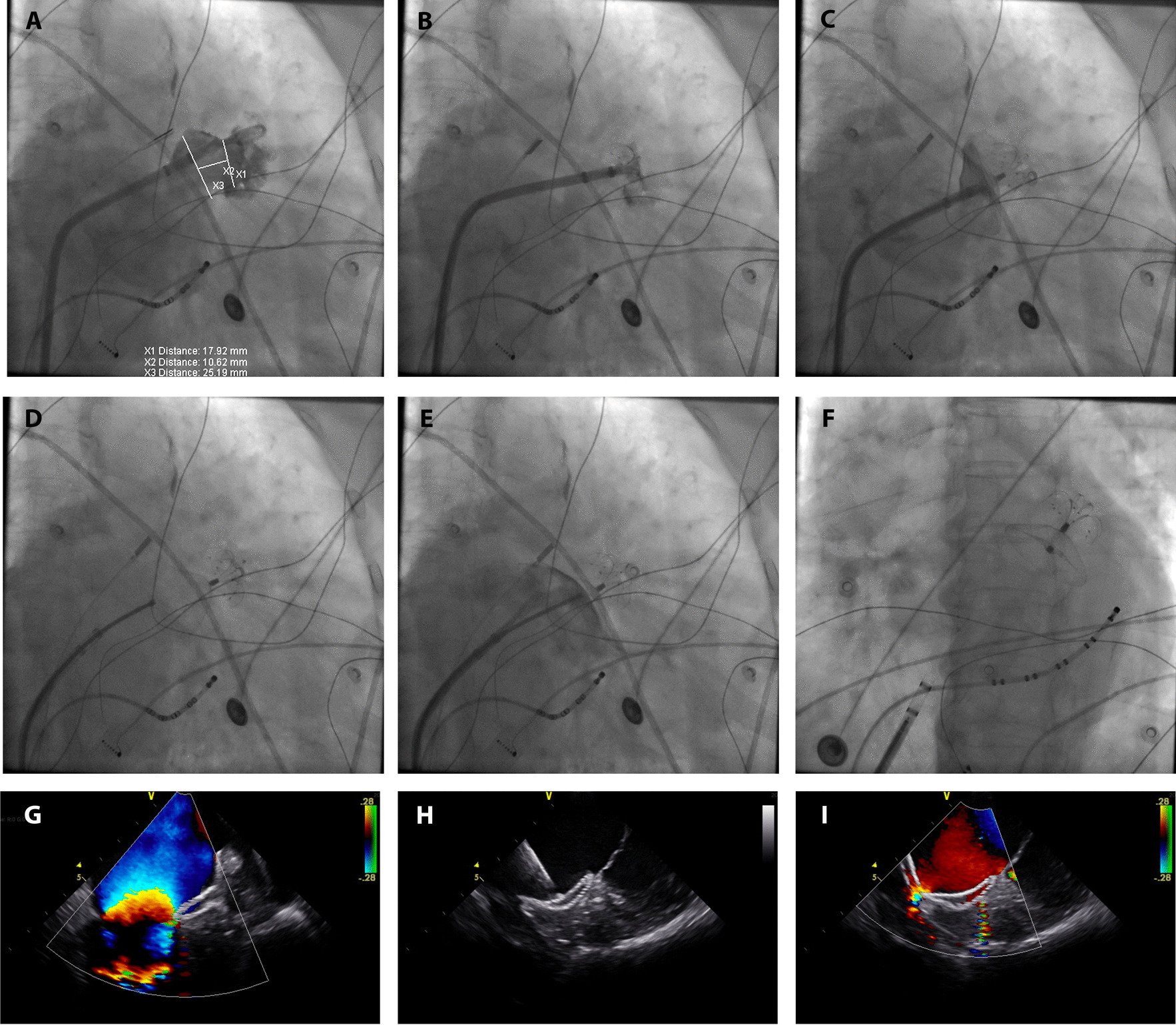
A step-by-step illustration of the implantation procedure. **A** LAA angiography. **B** Umbrella deployment. **C** Cover deployment. **D** Complete sealing of the LAA before release. **E** Tug test before final release. **F** Complete sealing of the LAA after final release of the device. **G** and **H** Intracardiac echocardiography showing LAA occlusion by the LAmbre device. **I** Intracardiac echocardiography showing no peri-device leak. LAA: left atrial appendage; X1: diameter of the landing zone; X2: length of the central flexible waist; X3: diameter of the LAA orifice

**Fig. 3:**
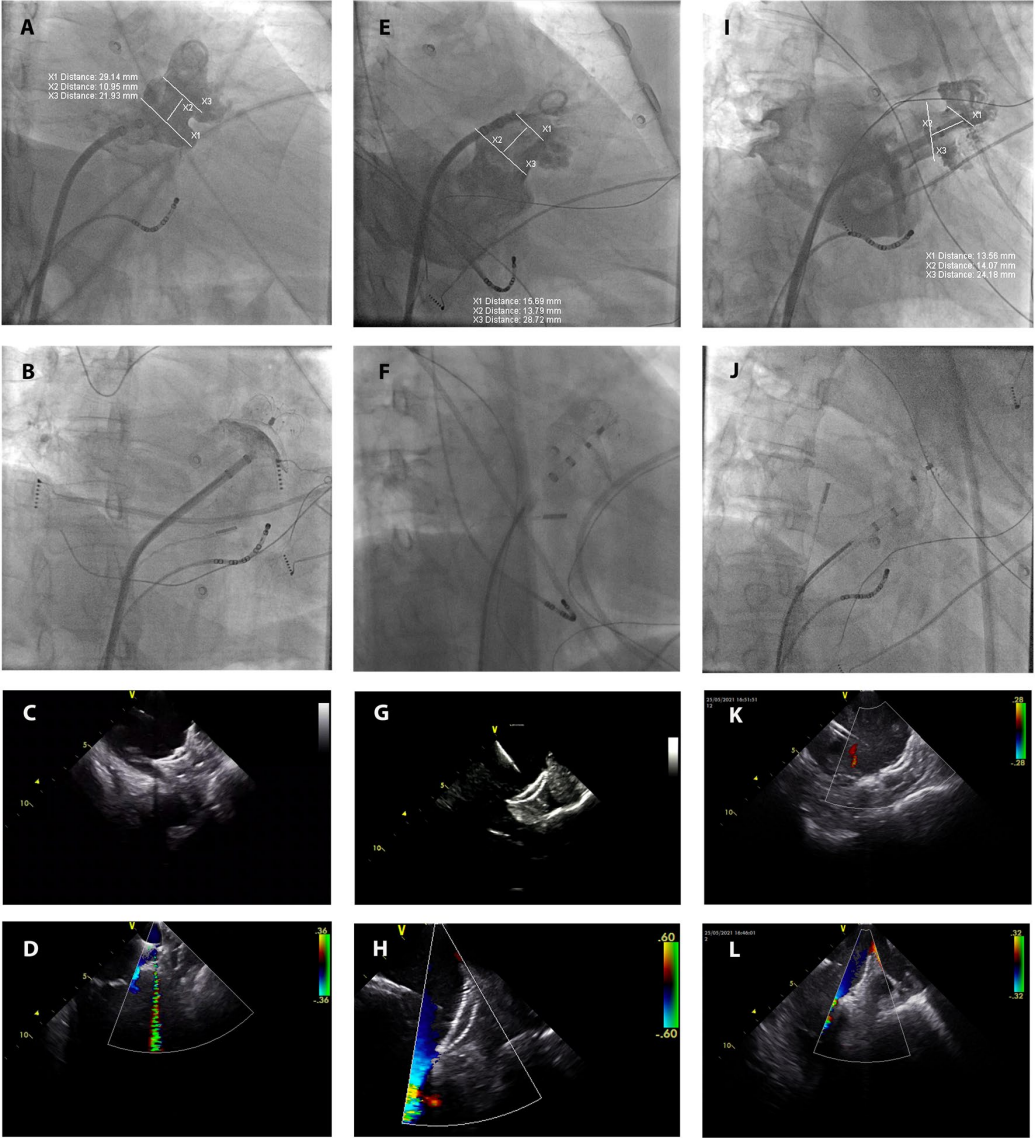
The small-umbrella LAmbre device anchored in the branch for multi-lobe LAA. **A–D** Case 1 using a small-umbrella LAmbre device. **E–H** Case 2 using a small-umbrella LAmbre device. **I–L** Case 3 using a small-umbrella LAmbre device. **A**, **E**, **I** LAA angiography. **B**, **F**, **J** Complete sealing of the LAA by the device. **C**, **G**, **K** Intracardiac echocardiography showing LAA occlusion by the LAmbre device. **D**, **H**, **L** Intracardiac echocardiography showing no peri-device leak. LAA: left atrial appendage; X1: diameter of the landing zone; X2: length of the central flexible waist; X3: diameter of the LAA orifice

**Fig. 4:**
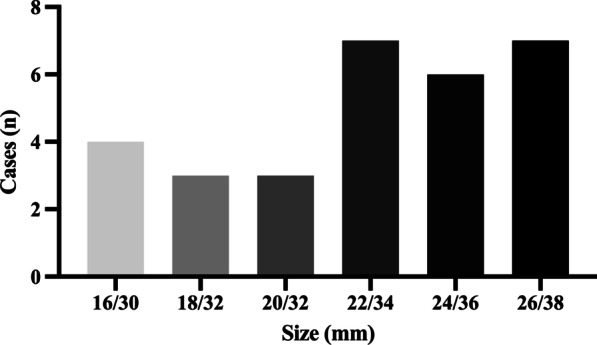
The size distribution of LAmbre devices

The small-umbrella LAmbre device (Fig. 1B and D) was successfully implanted in all 30 patients. A step-by-step explanation of the procedure is shown in Fig. 2. The procedural details are shown in Table 2. Two thirds of LAAs were cauliflower-type. Nearly three-quarters of patients (73.3%) had at least two lobes in the LAA. The inner umbrella of the device was anchored in the branch in 9 patients (30%), in the common trunks of branches in 13 patients, and in the main lobe in 8 patients with single-lobe LAA. The diameter of the LAA orifice was much greater than the LAA landing zone (27.4 ± 3.95 mm vs. 19.0 ± 4.39 mm, respectively) (Table 2). Accordingly, the mean diameters of the outer cover and inner umbrella of the occluder were 34.4 ± 2.75 mm and 22.0 ± 3.42 mm, respectively. Three of the 22 cases in which devices were anchored in the branch or common trunk are shown in Fig. 3. The distribution of different-sized occluders is shown in Fig. 4. The most frequently used sizes were 22/34 mm and 26/38 mm.

**Table 2:** Procedure details

LAA types	
Chicken wing	7 (23.3)
Windsock	2 (6.7)
Cactus	1 (3.3)
Cauliflower	20 (66.7)
LAA lobes	
1	8 (26.7)
2	20 (66.7)
≥3	2 (6.7)
Umbrella position in the LAA	
Branch	9 (30)
Common trunk of branches	13 (43.3)
Main lobe (one-lobe LAA)	8 (26.7)
Parameters of the LAA	
Diameter of LAA orifice, mm	27.4 ± 3.95
Diameter of LAA landing zone, mm	19.0 ± 4.39
Orifice/Landing zone ratio	1.6 ± 0.31
Difference between orifice and landing zone, mm	9.2 ± 4.19
Parameters of the LAmbre device	
Diameter of outer cover, mm	34.4 ± 2.75
Diameter of inner umbrella, mm	22.0 ± 3.42
Cover/Umbrella ratio	1.6 ± 0.14
Difference between cover and umbrella, mm	12.5 ± 0.86
Device selection and deployment	
Success at first device selected	21 (70.0)
Success at second device selected	6 (20.0)
Success at third device selected	3 (10.0)
Success at first deployment	14 (46.7)
Number of retrieve and re-deployment	1.4 ± 1.6
TEE guidance	18 (60.0)
ICE guidance	12 (40.0)
Peri-device leak	
No residual flow	26 (86.7)
Residual flow < 1 mm	4 (13.3)
Residual flow 1–3 mm	0
Residual flow > 3 mm	0

*ICE* intracardiac echocardiography, *LAA* left arial appendage, *TEE* transesophageal echocardiography. The TEE and ICE results presented are site reports and not core lab evaluations

Implantation was successfully performed with the first selected device in 21 patients (70.0%), and satisfactory occlusion was achieved at the first attempt in 14 patients (46.7%) (Table 2). The procedure was guided by TEE in 18 patients (60%) and by ICE in 12 patients (40%). No peri-device residual flow was detected in 26 patients (86.7%), and mild (< 1 mm) residual flow was detected in 4 patients (13.3%) after final release of the occluder (Table 2). There were no serious periprocedural complications (Additional file 1: Table 1). Mild pericardial effusion (≤ 3 mm) occurred in 3 patients, which disappeared within 48 h after the procedure, and none of them needed further interventions.

### Follow-up

At 3 months after the procedure, follow-up TEE was performed in 24 patients. No cases of peri-device residual flow were observed (Additional file 1: Table 2). Device thrombosis detected by TEE was reported in 1 patient at the 3-month follow-up. The thrombosis was on the surface of the device. The patient was treated with dabigatran after the procedure, and it was continued for another 3 months. At the 6-month follow-up by TEE, the thrombosis disappeared. Ischemic stroke occurred in 1 patient 1 month after the procedure. The functional deficit was mild with a National Institutes of Health Stroke Scale of 3. No thrombolysis was needed. This patient also had a prior history of stroke. No stroke events occurred during the following 1-year follow-up for this patient. At the 6-month and 1-year follow-up of all patients by cardiac CT, no peri-device residual flow or device-related thrombosis was reported. No death or hemorrhagic stroke events were reported during follow-up.

Within 3 months after the procedure, 4 patients were treated with warfarin, 5 with dabigatran, and 21 with rivaroxaban. Except for the 2 patients mentioned above, other 28 patients were treated with aspirin and clopidogrel from the third to sixth months after the procedure. All patients received long-term mono-antiplatelet therapy with aspirin 6 months after the procedure.

## Discussion

To our knowledge, this study is the first to explore the feasibility, safety, and efficacy of the small-umbrella LAmbre device for occlusion of morphologically complicated LAA. The results demonstrate that LAA occlusion using this occluder was feasible with no major procedural complications or short-term adverse events in this study. The high success rates of implantation and occlusion during the procedure and at the short-term follow-up indicate that this occluder could be easily adaptable to various types of LAA, in particular anatomically complex LAA.

All patients in this study were strictly screened for LAA occlusion indications according to current guidelines and expert consensus [1, 2, 10]. All patients in this study were at a high risk of stroke, 15 (50%) of whom had a history of stroke (2 had previously experienced stroke more than twice), and 7 of whom had a history of confirmed or suspicious LAA thrombosis. The CHA_2_DS_2_-VASc score was ≥ 2 (≥ 3 for females) in all patients, ≥ 3 in 23 patients (76.7%), and ≥ 5 in 13 patients (43.3%). In summary, 20 patients underwent LAA occlusion for a high bleeding risk (HAS-BLED score of ≥ 3), and 2 of them also experienced stroke events when taking adequate anticoagulation therapy with evidence of thrombosis originating from the LAA. The other 10 patients had documented poor adherence to anticoagulation and refused anticoagulation, even after personal and detailed advice (5 of these patients had a history of stroke).

### Unique features of the small-umbrella LAmbre device

**Fig. 5:**
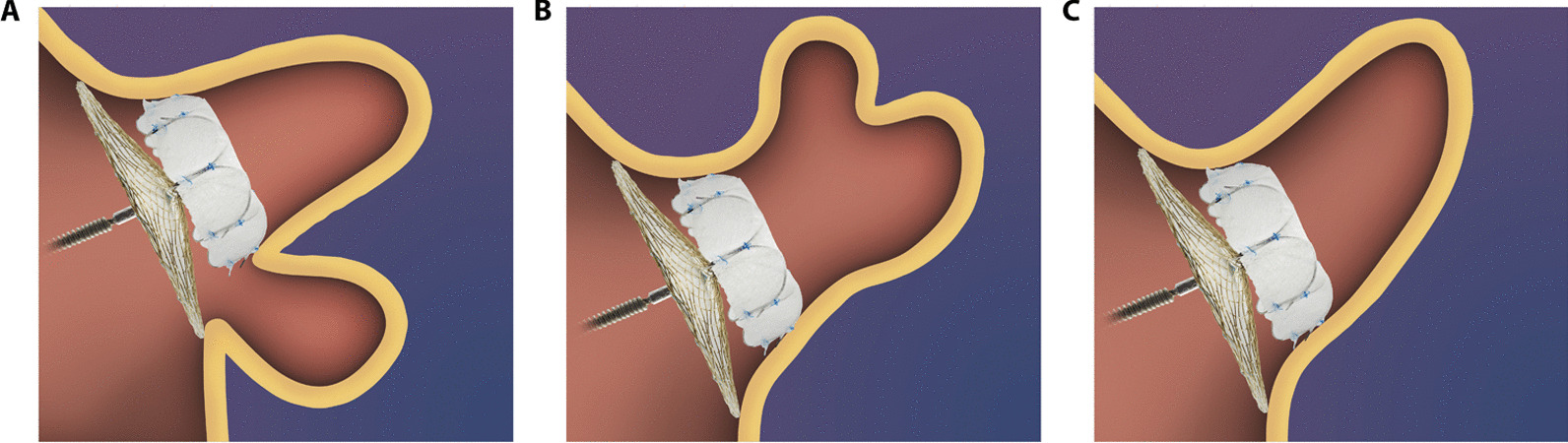
A schematic diagram of the application of the small-umbrella LAmbre device in various types of LAA. **A** Multiple-proximal-lobe LAA, with the inner umbrella anchored in the branch. This is the most ideal condition for the small-umbrella LAmbre device. **B** Multiple-distal-lobe LAA with a large orifice and the inner umbrella anchored in the common trunk. **C** Single-lobe LAA with a large orifice and the inner umbrella anchored in the main lobe. LAA: left atrial appendage

Currently, most approved LAA occlusion devices are designed for single-lobe LAA or anatomically regular LAA. However, an autopsy study showed that LAAs have diverse morphologies, and up to 80% of LAAs might have two or more lobes [24]. The LAmbre LAA occlusion device comes in 17 sizes, which makes this device highly adaptable to different LAA morphologies, and they can be quite useful for occlusion of LAA with a complex anatomy. The small-umbrella LAmbre device was mainly designed for multi-lobe LAA. The ideal application was to implant the inner umbrella in one of the branches and place the outer cover on the orifice of the LAA (Fig. 5A). However, from our clinical experience, we believe that this small-umbrella LAmbre device could be used mainly in three conditions: (1) multiple-proximal-lobe LAA (Fig. 5A); (2) multiple-distal-lobe LAA with a large orifice (Fig. 5B); and (3) single-lobe LAA with a large orifice (Fig. 5C). In these conditions, the landing zone of the umbrella could be the branch (Fig. 5A), the common trunk (for multi-lobe LAA) (Fig. 5B), or the main lobe (for single-lobe LAA) (Fig. 5C). These LAAs share one common feature, which is the relatively large orifice compared with the lobe or the common trunk. In this study, the mean diameters of the LAA orifice and landing zone were 27.4 mm and 19.0 mm, respectively, with a ratio of 1.6, which was significantly greater than that in previous studies (23.6/22.7 mm in the study by Huang et al. [15], and 22.7/21.1 mm in the study by Park et al. [20]. Hence, in these patients, a cover that was relatively larger than the umbrella was needed. In this study, the difference in the diameter between the cover and the umbrella was at least 12 mm (Fig. 4). This special design is the key element for appropriate and safe anchoring of the small umbrella in the landing zone and thorough sealing of the large cover on the LAA orifice. Therefore, this study proved that the small-umbrella LAmbre device could be well adapted in morphologically complicated LAAs (Fig. 5).

Theoretically, with multiple-distal-lobe LAA with a large orifice (Fig. 5B) and single-lobe LAA with a large orifice (Fig. 5C), larger plug-type LAA occlusion devices could also be used, while traditional pacifier-type devices with regular sizes might not be suitable.

### Feasibility, safety, and short-term efficacy of the small-umbrella LAmbre device

Implantation was successful in all 30 patients, supporting the feasibility of the small-umbrella LAmbre device. Due to the relatively complicated anatomy of the LAA, 9 patients (30%) required device size reselection, and 16 patients (53.3%) required device repositioning. However, only 3 patients demonstrated pericardial effusion after the procedure, with no other major or minor procedural complications. Hence, the small-umbrella occluder was safe compared with regular-sized devices [15].

During the procedure, only 4 patients had minimal peri-device residual flow (< 1 mm), indicating a 100% acute sealing rate. Of the 24 patients examined by TEE 3 months after the procedure, none had peri-device residual flow, suggesting a high short-term sealing rate. Therefore, the small-umbrella LAmbre device effectively sealed morphologically complicated LAA.

LAA occlusion in 12 patients (40%) was guided by ICE. In these patients, the procedure could be performed under local anesthesia. ICE could also be advanced into the left atrium to obtain a better view of the LAA and the occlusion device than TEE. This could shorten the procedure time, reduce anesthesia-related complications, and guarantee a better sealing effect.

### Adverse events during follow-up

One patient experienced stroke at 1 month after the procedure, although contrast-enhanced cardiac CT and TEE suggested that the LAA was perfectly sealed. This patient might have had non-cardiac reasons for stroke. One patient demonstrated thrombosis formation on the device at the 3-month TEE follow-up, and adequate anticoagulation was prescribed for 3 months. The thrombosis was resolved at the 6-month follow-up TEE. No other adverse events were reported, including death, hemorrhagic stroke, systemic thromboembolism, device dislocation, or serious pericardial effusion. Overall, the adverse event rate was low during short-term follow-up. The absence of tamponade and device dislodgement is due to the unique design of LAmbre, in which the umbrella behaves like a balloon expandible stent, and once fully expanded, no further radial force is continuously affecting the LAA wall.

### Limitations

This study has several limitations. First, it was a retrospective study, and no control groups, such as groups in which other devices were used for LAA occlusion, were included. Second, the sample size was relatively small, which might be due to the strict enrollment criteria for LAA occlusion and the relatively low rate of anatomically complicated LAA in patients with indications. However, this is the largest study reporting the use of the small-umbrella LAmbre device for LAA occlusion. Third, this study focused on evaluating the peri-procedural and short-term safety and efficacy outcomes after LAmbre device implantation. A long-term follow-up study is currently ongoing. The favorable outcomes in the short-term and the special design of this device suggest a promising outcome in the long-term. Fourth, implantation of the small-umbrella LAmbre device designed for morphologically complicated LAA might require greater operator experience.

## Conclusions

Occlusion of morphologically complicated LAA using the small-umbrella LAmbre device was feasible, safe, and effective in patients with NVAF in this study. The small-umbrella occluder provides an alternative for patients who are not optimal candidates for regular-sized LAA occlusion devices.

## Supplementary Information

**Additional file 1.** Supplemental Material.

## Data Availability

Research data is confidential. Data sharing requests are required to meet the policies of the hospital and the funder. Please contact Dr. Min TANG (Email: doctortangmin@yeah.net) for Research data.

## Abbreviations

AF: Atrial fibrillation
NVAF: Non-valvular AF
TEE: Transesophageal echocardiography
LAA: Left atrial appendage
ICE: Intracardiac echocardiography
CT: Computed tomography
