# Supplementary material for: Occluding morphologically complicated left atrial appendage using the small-umbrella LAmbre device
Source: BMC Cardiovasc Disord. 2022 Jul 23;22:326. doi: 10.1186/s12872-022-02770-y (PMC9308203; doi:10.1186/s12872-022-02770-y)
Supplement: Supplementary file 1 — Additional file 1. Supplemental Material. [file 12872_2022_2770_MOESM1_ESM.docx]

Supplemental Material

Supplemental Table 1. Procedure complications

| Major complications |  |
| --- | --- |
| Death | 0 |
| Pericardia effusion requiring intervention | 0 |
| Stroke | 0 |
| Major bleeding | 0 |
| Device dislocation | 0 |
| Minor/Access vessel complications |  |
| Femoral hematoma | 0 |
| Arteriovenous fistula | 0 |
| Pseudoaneurysm | 0 |
| Mild Pericardial Effusion | 3 |

Supplemental table 2. Short-term Outcomes

| Adverse events |  |
| --- | --- |
| All cause Death | 0 |
| Cardiac or unexplained death | 0 |
| Non-cardiac death | 0 |
| Ischemic stroke | 1 |
| Hemorrhagic stroke | 0 |
| Systemic thromboembolism | 0 |
| Thrombosis formation on the device | 1 |
| Device dislocation | 0 |
| Serious Pericardial effusion | 0 |
| LAA sealing by TEE examination |  |
| No residual flow | 24 |
| Residual flow < 1 mm | 0 |
| Residual flow 1-3 mm | 0 |
| Residual flow > 3 mm | 0 |
